# Multilocus microsatellite typing of *Leishmania infantum* isolates in monitored *Leishmania/*HIV coinfected patients

**DOI:** 10.1186/s13071-015-0989-9

**Published:** 2015-07-22

**Authors:** Míriam Tomás-Pérez, Mallorie Hide, Cristina Riera, Liliana Montoya, Anne-Laure Bañuls, Esteve Ribera, Montserrat Portús, Roser Fisa

**Affiliations:** Laboratory of Parasitology, Faculty of Pharmacy, Universitat de Barcelona, Avda Joan XXIII s/n, 08028 Barcelona, Spain; MIVEGEC, UMR IRD 224-CNRS 5290-Université de Montpellier, Montpellier, France; Infectious Diseases Department and Microbiology Department, Hospital Universitari Vall d’Hebron, Universitat Autònoma de Barcelona, Barcelona, Spain

**Keywords:** *Leishmania infantum*, HIV+, Microsatellites, Genetic diversity, Phenetic analyses, Relapse, Reinfection

## Abstract

**Background:**

*Leishmania infantum* is the main etiological agent of both visceral and cutaneous clinical forms of leishmaniasis in the Mediterranean area. *Leishmania*/HIV coinfection in this area is characterized by a chronic course and frequent recurrences of clinical episodes. The present study using Multilocus Microsatellite Typing (MLMT) analysis, a highly discriminative tool, aimed to genetically characterize *L. infantum* isolates taken from monitored *Leishmania*/HIV coinfected patients presenting successive clinical episodes.

**Methods:**

In this study, by the analysis of 20 microsatellite loci, we studied the MLMT profiles of 25 *L. infantum* isolates from 8 *Leishmania*/HIV coinfected patients who had experienced several clinical episodes. Two to seven isolates per patient were taken before and after treatment, during clinical and non-clinical episodes, with time intervals of 6 days to 29 months. Genetic diversity, clustering and phenetic analyses were performed.

**Results:**

MLMT enabled us to study the genetic characteristics of the 25 *L. infantum* isolates, differentiating 18 genotypes, corresponding to a genotypic diversity of 0.72. Fifteen genotypes were unique in the total sample set and only 3 were repeated, 2 of which were detected in different patients. Both clustering and phylogenetic analyses provided insights into the genetic links between the isolates; in five patients isolates showed clear genetic links: either the genotype was exactly the same or only slightly different. In contrast, the isolates of the other three patients were dispersed in different clusters and some could be the result of mixing between populations.

**Conclusions:**

Our data indicated a great MLMT variability between isolates from coinfected patients and no predominant genotype was observed. Despite this, almost all clinical episodes could be interpreted as a relapse rather than a reinfection. The results showed that diverse factors like an intrapatient evolution over time or culture bias could influence the parasite population detected in the patient, making it difficult to differentiate between relapse and reinfection.

## Background

The first case of leishmaniasis associated with HIV infection was reported in 1985. Since then, cases of coinfection have been reported in 35 countries around the world, with an increasing number occurring in southern Europe. The four European countries most affected are France, Italy, Portugal and above all Spain, the latter having the highest incidence due to a greater geographical overlap between leishmaniasis and HIV infections [1]. *Leishmania infantum* is the main etiological agent of both visceral and cutaneous clinical forms of human leishmaniasis in this area, which is transmitted through vectors of the species *Phlebotomus perniciosus* and *P. ariasi* [2]. These cases of leishmaniasis/HIV coinfection usually result in visceral leishmaniasis (VL), and are often thought to be related to the reactivation of asymptomatic infections [1]. Acquired infection among intravenous drug users (IVDU) sharing contaminated syringes has also been reported, which could represent an anthroponotic cycle [3]. VL cases associated with HIV are characterized by the appearance of numerous recurrences of clinical episodes that vary in number and average duration according to the immunological status of patients, parasite species, and use of anti-leishmanial therapy [1].

For the epidemiological study and follow-up of these coinfection cases, highly discriminatory methods are needed to differentiate *Leishmania* at the strain level [4, 5]. Previous research on different *Leishmania* strains of coinfected patients in France using isoenzymatic characterization (MLEE) revealed a low polymorphism in primo infection as well as in relapse cases [6]. Research performed in Catalonia, our area of study, has also registered a low polymorphism among coinfected patients, with more than 50 % of the studied strains belonging to zymodeme MON-1 [7]; in the south of Spain, however, a greater polymorphism was found by MLEE studies, although almost 50 % of strains were described as zymodeme MON-1 [8, 9].

Microsatellite markers have proved to be powerful tools for molecular typing and population genetic studies in *Leishmania*, being able to discriminate among zymodemes, even within MON-1 [4]. Microsatellites are short nucleotide fragments of 1 to 6 bp repeated in tandem and ubiquitously distributed in the genomes of eukaryotic organisms [10]. They present high rates of mutation and variability due to allelic repeat length variation [11, 12]. The Multilocus Microsatellite Typing (MLMT) approach developed for the *Leishmania donovani* complex is based on a set of 14–20 unlinked microsatellite loci [12]. The present MLMT study aimed to genetically characterize *L. infantum* isolates taken from monitored *Leishmania*/HIV coinfected patients presenting subsequent clinical episodes. The specific objective was to define the genetic links between the parasites of VL patients under study, and between parasites from repeated isolates from the same patient. Besides describing a *Leishmania* population in HIV patients, this genetic study attempted to generate data that would help to differentiate between parasite reinfections and relapses.

## Methods

### Patients

The study included 8 adults (7 men and 1 woman) with *Leishmania*/HIV coinfection from the Barcelona metropolitan area, monitored at the Hospital Vall d’Hebron (Barcelona, Spain). Six patients were IVDU, aged from 27 to 44 years at the start of the study, when the first *Leishmania* isolate was obtained (Table 1).

**Table 1:** Identification of the 25 *Leishmania* isolates used in this study from 8 *Leishmania*/HIV coinfected patients obtained during VL clinical episodes or non-clinical episodes, from bone marrow or peripheral blood mononuclear cell samples; and their genotyping and clustering analysis results

Patient code	Patient isolates	Sex	Age	Risk factor	Time from first episode (in months)	Clinical status	Sample Type	WHO Code	Genotype (G)	Cluster (C)
P1	1a	Man	30	IVDU	June	CE	PBMC	MHOM/ES/00/BCN-278	G14	C2b
	1b				8 m	CE	PBMC	MHOM/ES/01/BCN-376	G13	C2b
	1c				20 m	CE	PBMC	MHOM/ES/02/BCN-464	G15	C2b
P2	2a	Man	44	IVDU	July	CE	BM	MHOM/ES/00/BCN-284	G3	C1
	2b				2 m	CE	BM	MHOM/ES/00/BCN-289	G2	C1
P3	3a	Man	36	nd	May	CE	BM	MHOM/ES/01/BCN-404	G16	C2
	3b				7 m	NE	PBMC	MHOM/ES/01/BCN-455	G16	C2
	3c				12 m	CE	BM	MHOM/ES/02/BCN-492	G16	C2
P4	4a	Man	34	IVDU	April	CE	BM	MHOM/ES/02/BCN-475	G9	C2
	4b				6 m	CE	BM	MHOM/ES/02/BCN-508	G9	C2
P5	5a	Man	36	IVDU	September	CE	BM	MHOM/ES/94/BCN-123	G10	C2
	5b				5 m	CE	BM	MHOM/ES/95/BCN-130	G1	-
P6	6a	Man	33	IVDU	October	CE	BM	MHOM/ES/00/BCN-298	G11	C2
	6b				6 m	CE	BM	MHOM/ES/01/BCN-400	G5	C2
	6c				17 m	CE	BM	MHOM/ES/02/BCN-470	G12	C2
P7	7a	Man	41	nd	October	CE	PBMC	MHOM/ES/01/BCN-430	G7	C2a
	7b				3 m	NE	BM	MHOM/ES/02/BCN-460	G7	C2a
	7c				4 m	CE	PBMC	MHOM/ES/02/BCN-472	G6	C2a
P8	8a	Woman	27	IVDU	October	CE	PBMC	MHOM/ES/00/BCN-293	G4	C2
	8b				1 m	NE	PBMC	MHOM/ES/00/BCN-306	G7	C2a
	8c				1 m + 6 days	NE	PBMC	MHOM/ES/01/BCN-307	G8	C2
	8d				3 m	NE	PBMC	MHOM/ES/01/BCN-369	G16	C2
	8e				7 m	CE	PBMC	MHOM/ES/01/BCN-405	G18	C2
	8f				9 m	NE	PBMC	MHOM/ES/01/BCN-422	G17	C2
	8 g				29 m	NE	PBMC	MHOM/ES/03/BCN-561	G16	C2

*IVDU* intravenous drug user, *nd* not determined, *CE* clinical episode, *NE* non-clinical episode, *PBMC* peripheral blood mononuclear cell, *BM* bone marrow

All patients were under HAART therapy (Highly Active AntiRetroviral Therapy). Diagnosis of VL was confirmed by culture in bone marrow or peripheral blood. After diagnosis, patients received one of the following treatments at standard doses: liposomal amphotericin B or amphotericin B lipid complex.

### Ethical approval

The study protocol was approved by the institutional review board of the hospital, and all patients signed informed consent for their participation in this study.

### *Leishmania* isolates

Twenty-five isolates were obtained from clinical and non-clinical episodes of the 8 patients. Two to seven isolates per patient were taken before and after treatment, with time intervals of 6 days to 29 months (Table 1). Eighteen isolates were obtained during clinical episodes and seven during non-clinical episodes. “*In vitro”* culture was performed using NNN (Novy-McNeal-Nicolle’s) medium and/or Schneider's insect culture medium (Sigma, St. Louis, MO) supplemented with 20 % heat-inactivated fetal calf serum, 1 % sterile human urine, and 25 μg/mL gentamicin solution. Cultures were maintained between 24 °C and 26 °C, examined twice a week, and sub-cultured every 2 weeks for 6 months before being considered negative. All isolates were stored in the *Leishmania* Cryobank at the Universitat de Barcelona. A *L. infantum* strain from Toulouse (France) was used as a reference to build the neighbor-joining (NJ) tree: LEM2355 (WHO code: MHOM/FR/91/LEM2355; MON-183).

### DNA extraction

One cryovial from each isolate was used for isolation of DNA. The promastigotes were quickly thawed and DNA was extracted using a chelex resin protocol: 100 μL of sterile water and 400 μL of chelex solution [1 % Tween 20 (Sigma, St. Louis, MO), 1 % Nonidet P-40 (Sigma, St. Louis, MO) and 20 % of chelex resin (BioRad Laboratories, Hercules, CA)] were added to the promastigote sediment. It was heated at 100 °C for 20 min and then vortexed. Finally, the mixture was centrifuged for 10 min at 12,000 g to separate the resin and the supernatant was collected as the substrate for the PCR, either performed immediately or after storage at −20 °C. All isolates were confirmed as *L. infantum* by specific PCR [13, 14].

### Multilocus Microsatellite Typing (MLMT)

The genotyping was done using 20 microsatellite markers previously described for the genetic characterization of *L. infantum* (Table 2). Amplification was performed in a volume of 30 μL containing 3 μL of 10X buffer, 1 nmol of dNTP mix, and 10 pmol of each primer (the forward being labeled) and 1.5 units of Taq polymerase (Taq Polymerase, 5U/μL, Roche Diagnostics, France). We added 50 ng of extracted DNA to the mixture and incubated it in a thermal cycler under the following conditions: a denaturing step at 94 °C for 2 min, followed by 35 cycles of denaturation for 30 s at 94 °C, annealing for 1 min at the annealing temperature of each locus (Table 2) and extension for 1 min at 72 °C, followed by a final extension at 72 °C for 30 min. The amplified products were analyzed using an automated fragment analysis on an ABI Prism 3130XL genetic analyzer (Applied Biosystems, France) with a Genescan 500 LIZ internal size standard. Finally, data were analyzed with GeneMapper analysis software (version 4.0, Applied Biosystems, France).

**Table 2:** Characteristics of the 20 microsatellite loci used in this study for *Leishmania infantum* genotyping

Locus	GenBank accession no.	Allele size (bp)	Dye label	*Ta* (°C)	*Na*	*H*_*S*_	*H*_*o*_
Li22-35^c^	AM050045	90–106	VIC	58	5	0.191	0.042
Li45-24^c^	AM050048	88–108	NED	58	5	0.208	0.060
Li71-5/2^c^	AM050050	104–108	VIC	54	3	0.280	0.042
Li72-20^c^	AM050057	87–95	VIC	50	4	0.275	0.042
LiBTA^a^	nd	226–246	VIC	58	4	0.316	0.167
LiBTG^a^	nd	219–257	6-FAM	58	7	0.340	0.104
LIST7021^b^	AF427869	228–246	6-FAM	54	6	0.338	0.143
LIST7024^b^	AF427872	198–224	VIC	59	3	0.049	0.000
LIST7025^b^	AF427873	171–179	6-FAM	56	1	0.000	0.000
LIST7026^b^	AF427874	201–231	NED	56	4	0.089	0.000
LIST7028^b^	AF427876	104–108	VIC	58	2	0.049	0.000
LIST7031^b^	AF427879	166–174	PET	54	1	0.000	0.000
LIST7033^b^	AF427881	196–226	6-FAM	58	5	0.349	0.411
LIST7035^b^	AF427883	188–202	PET	56	6	0.373	0.146
LIST7037^b^	AF427885	178–194	6-FAM	58	5	0.320	0.411
LIST7038^b^	AF427886	122–130	NED	56	3	0.088	0.000
LIST7039^b^	AF427887	199–215	PET	58	5	0.128	0.143
Rossi1^d^	X76394	104–110	6-FAM	59	3	0.100	0.000
Rossi2^d^	X76393	140–160	VIC	57	6	0.335	0.185
TubCA^c^	nd	74–84	6-FAM	58	2	0.100	0.000
Mean value					4	0.196	0.095

*Ta*: annealing temperature (thermocycling conditions); *Na*: number of alleles; Hs: Nei’s unbiased genetic diversity within subsamples; Ho: observed heterozygosity (Nei & Chesser, 1983 [17]); ^a^Bulle, 2002 [32]; Hide, 2013 [24]; ^b^Jamjoon, 2002 [33]; ^c^Ochsenreither, 2006 [11]; ^d^Rossi, 1994 [34]

### Genetic diversity and differentiation analysis

Data were analyzed with FSTAT Version 2.9.3.2 [15] updated from [16], which allows the calculation of diversity indices such as Nei’s unbiased genetic diversity index within sub-samples (*H*s), the observed heterozygosity (*H*o), as a measure of genetic diversity [17], and the number of alleles per locus (*N*a) measuring genetic polymorphism. The genotypic diversity was calculated as the ratio of the number of genotypes per total number of samples.

### Clustering and phenetic analyses

The genetic characteristics of the *Leishmania* samples under study were investigated with MLMT data by two different methods. The first was based on genetic distances by the construction of a phenetic tree according to the proportion of shared allele distances (D_AS_). The Neighbor-Joining (NJ) tree [18] was constructed through calculations of Cavalli-Sforza genetic distance from allelic frequencies, and the robustness of tree topology was obtained by bootstrap resampling of loci, with 100 replications per set. We used PHYLIP software (3.67 package; J. Felsenstein, 1993. Department of Genetics, University of Washington, Seattle, USA) and the tree was edited and visualized with TreeDyn software [19]. The second approach consisted of a model-based Bayesian clustering method implemented in STRUCTURE v 2.3.1 [20]. This algorithm simultaneously estimates the allele frequencies to assign individuals into genetically distinct populations (*K*) and each probability for the identification of the most likely number of populations. The allele frequencies among populations were correlated by admixture modeling for a series of runs using a ‘burn-in’ period of 20,000 iterations and probability estimates based on 200,000 of Markov chain Monte Carlo (MCMC) repeats. Ten independent runs for each *K* were carried out for each possible number of clusters (*K*) in order to quantify the variation in the likelihood of the data for a given *K*. The most appropriate number of populations was determined based upon *ad hoc* statistic ∆*K*, which evaluates the second order rate of change of the likelihood function with respect to the number of populations *(K*).

## Results

### Genetic diversity

All 20 microsatellite *loci* used for the analysis of the 25 *L. infantum* isolates produced clear electrophoregrams, with only one or two alleles at each locus. We found 18 polymorphic microsatellite markers, with LiBTG as the most polymorphic, having 7 different alleles (*N*_a_) and two monomorphic markers, LIST7025 and LIST7031. We observed 8 polymorphic microsatellites with more than 5 alleles, LIST7021, LIST7035 and Rossi2 with 6 alleles each; and Li22-35, Li45-24, LIST7033, LIST7037 and LIST7039 with 5 alleles. Markers Li72-20, LiBTA and LIST7026 revealed 4 alleles; Li71-5/2, LIST7024, Rossi1 and LIST7038 3 alleles; TubCA and LIST7028 showed 2 alleles. The mean value was 4 alleles per locus (Table 2). The diversity analysis, including all the 25 isolates, revealed an observed heterozygosity (*H*_o_) between 0 and 0.411 (overall 0.095); the mean intrapopulation genetic diversity (*H*_s_) ranged between 0 and 0.373, with an overall value of 0.196 (Table 2).

### Genotype analysis and phylogenetic reconstruction

A total of 18 genotypes (G) were characterized for the 25 isolates belonging to the 8 monitored patients (Table 1), corresponding to a genotypic diversity of 0.72. Of these genotypes, 15 were unique in the total sample set, and only 3 (G7, G9 and G16) were repeated. The three repeated genotypes were found in four different patients: G7 in two of the three isolates of patient P7 and one of the seven isolates of patient P8; G9 in the two isolates of patient P4; and G16 in the three isolates of patient P3 and two isolates of patient P8. Only genotypes G16 and G7 were detected in two different patients.

**Fig. 1:**
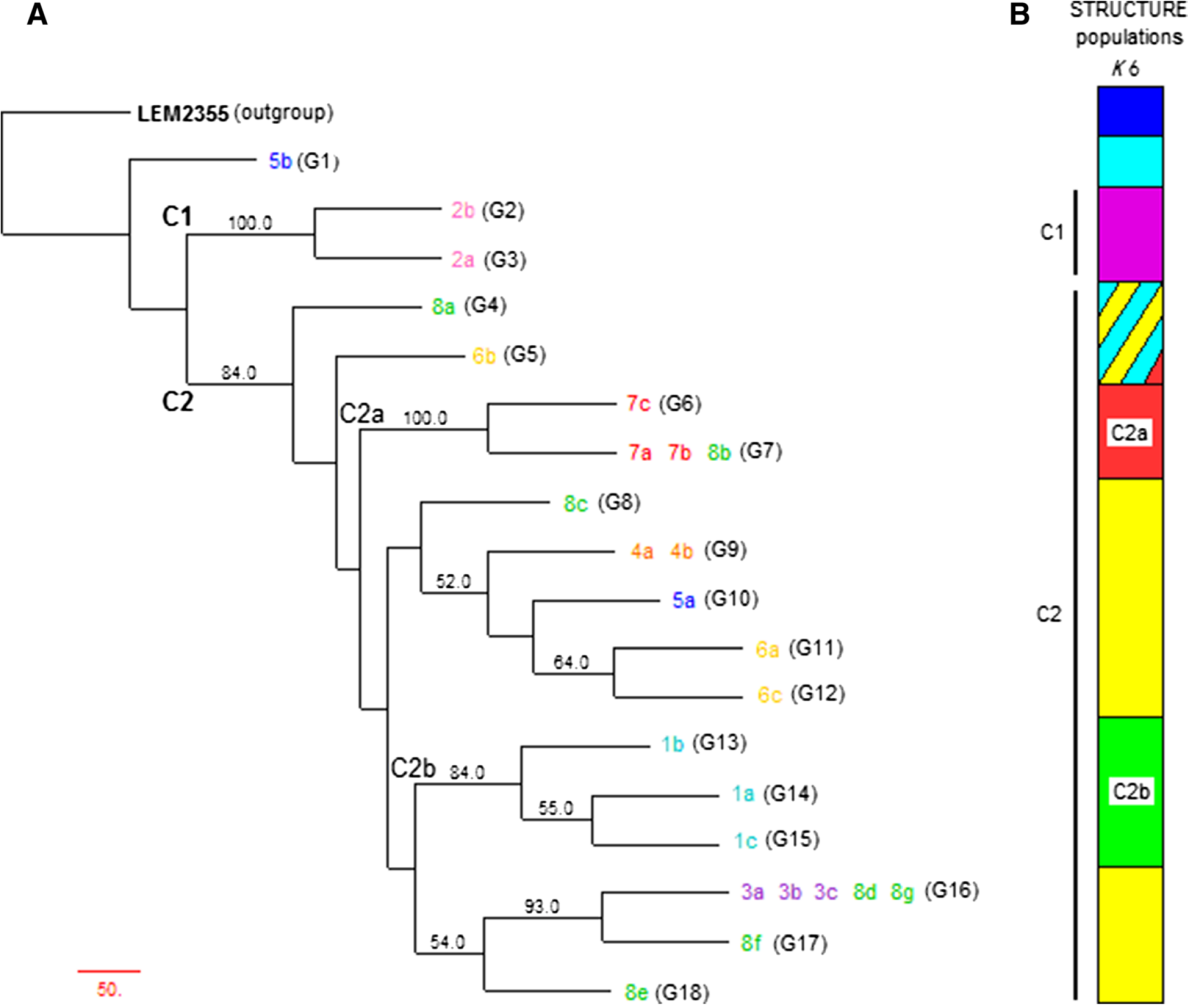
Phylogenetic and clustering analysis of 25 *L. infantum* isolates from 8 *Leishmania*/HIV coinfected patients for the data of 20 microsatellite markers. **a** Neighbor-joining tree inferred from the Cavalli-Sforza genetic distance results using PHYLIP and TreeDyn software for tree edition and visualization. The bootstrap values above 50 % are shown. The *Leishmania* isolates number (in colors), genotypes (G1-G18), clusters (C1 and C2) and sub-clusters (C2a and C2b) are indicated. **b** Populations identified by STRUCTURE (K = 6) divided in colors, organized according to the neighbor-joining tree. Clusters and sub-clusters are also indicated

MLMT profiles were used to calculate genetic distances and build a neighbor-joining tree. The genetic distance tree allowed us to differentiate 2 main populations or clusters C1 and C2, the latter composed of 2 well-supported sub-clusters C2a and C2b (Fig. 1). The reference strain LEM2355 was used as the outgroup. At the top of the tree, isolate 5b from patient P5 is clearly separated from the others. The first cluster (C1) is composed of the two isolates obtained from patient P2, separated from the others with a strong bootstrap value of 100 %. The second cluster (C2) is defined by a bootstrap value of 84 % and contains all the other 22 isolates. In C2 we can detect several sub-clusters, but only two, representing 5 samples and designated as C2a and C2b, are supported by bootstrap values higher than 80 %. C2a, with a bootstrap value of 100 %, is composed of the three isolates of patient P7 and one isolate (8b) of patient P8; C2b is supported by a bootstrap value of 84 % and includes all isolates from patient P1.

### Clustering analysis

**Fig. 2:**
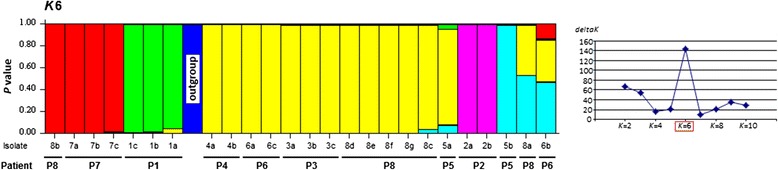
Population structure of the 25 *L. infantum* isolates coming from *Leishmania*/HIV coinfected patients as inferred by STRUCTURE software on the basis of 20 microsatellite data. The peak at *K* = 6 represents the most probable number of populations; vertical bars represent each analyzed isolate divided into *K* colors. Each color represents one population, and the length of the colored segments shows the estimated proportion of membership of the isolate in that population

The population organization of the 25 isolates was analyzed with STRUCTURE software for a better visualization of the data. Using the methods of Evanno and Garnier [21, 22], the analysis indicated the existence of 6 different populations (*K* = 6) (Figs. 1 and 2). Considering *K* = 6, all isolates from patients P2 and P1 defined two of these six populations, corresponding to the cluster C1 and the sub-cluster C2b of the phenetic tree. A third STRUCTURE population was defined by all isolates from patient P7 and one isolate from patient P8 (8b), corresponding to the sub-cluster C2a. A fourth population included all other isolates from C2, which were all isolates from patients P3 and P4, and some isolates belonging to patients P5, P6 and P8 (5a, 6a, 6c, 8c, 8d, 8e, 8f and 8 g). The two other populations were defined by the outgroup and one isolate from patient P5 (5b). The STRUCTURE analysis did not allow the classification of two isolates, 8a and 6b, which appear as mixed genotypes of NJ tree populations, C2, C2a and the non-classified isolate 5b from patient P5.

## Discussion

*L. infantum* is the causative species of VL and CL in the northwest Mediterranean area and an opportunistic parasite in HIV patients. It appears that immunocompromised people may be vulnerable to parasites that either fail to survive or never cause detectable morbidity in immunocompetent people. Furthermore, “dermotropic” variants of *L. infantum* have been reported to cause visceral disease in HIV-positive patients [1, 8, 23]. Other studies have revealed differences in parasitic genotypes between *L. infantum* strains from asymptomatic carriers and HIV-positive VL patients, suggesting that some genotypes do not cause disease [24]. Greater knowledge about the intraspecific variability of the *Leishmania* parasite from HIV-positive VL patients may help to understand some important key points, such as transmission patterns, response to treatment and the importance of immunity, and the parasite’s capacity to survive within human hosts.

In the present study, MLMT characterization of 25 *L. infantum* isolates showed the existence of a genetic polymorphism, with a mean number of alleles per locus (*N*_a_) of 4. Considering that our sample set came from only 8 coinfected patients monitored over time, from the restricted geographical area of Barcelona, the *N*_a_ is not negligible when compared with previous studies performed in nearby areas. In the South of France, a mean *N*_a_ of 4.13 was found within *L. infantum* isolates from symptomatic humans and dogs and asymptomatic humans [24]. A similar mean *N*_a_ value of 4.57 was obtained in Portugal from human, dog, vulpine and phlebotomine *L. infantum* isolates [25]. Likewise, another study performed on MON-1 *L. infantum* isolates from human patients, dogs and phlebotomine sand flies of Spain, Portugal and Greece obtained a mean *N*_a_ value of 4.6 [4].

Furthermore, 18 different genotypes were found in the 25 analyzed isolates, with a low number of repeated genotypes, despite their limited geographical and human origin. Fifteen genotypes were unique in the total sample set, and only three (G7, G9 and G16) were repeated; both G7 and G16 appeared in two different patients, and G9 in two isolates from the same patient. No relationship was observed between genotypes and the clinical status of patients, with 16 different genotypes among the 18 clinical episodes, and two (G7 and G16) of the three repeated genotypes present in clinical and non-clinical episodes. The kind of sample used in the study, bone marrow or peripheral blood, was not associated with particular genotypes either, as previously described by other authors [26].

Our data indicated a high degree of heterogeneity and no predominant genotype in the isolates, but a more extensive study is required to assess the real variability and abundance of genotypes in immunocompromised patients in this area. The parasite heterogeneity observed in our patient set is in agreement with the genetic variability described in a previous MLMT study on *Leishmania* isolates from dogs and sand flies performed in a rural leishmaniasis-endemic area close to Barcelona (Priorat, Tarragona) [26]. Other MLMT studies performed on *Leishmania* parasites from different hosts, geographical regions and clinical forms have also found a low number of repeated multilocus genotypes [25, 26]. A Portuguese study reported a generally low percentage (12 %) of repeated genotypes, although a higher percentage was found in coinfected patients [25], which was related to human-to-human transmission, notably associated with IVDU [27, 28]. The genotypic diversity registered in our study was 0.72, which is higher than in other studies performed in Europe, for example, 0.55 in Greece [29] or 0.67 in Portugal [25]. However, any comparison between these results is limited by the variable number of isolates and loci analyzed.

*Leishmania*/HIV coinfection is characterized by frequent recurrences of clinical episodes. Previous studies with monitored *L. infantum*/HIV coinfected patients in our endemic area revealed the existence of a residual parasite load, using qPCR analysis after patient treatment, which has been related to the chronic course of the disease and subsequent recurrences of VL [30]. It is fair to assume that, in general, clinical recurrences may be related to relapses produced by the same parasite rather than reinfections. Nevertheless, detection by qPCR did not allow parasite characterization to differentiate between a relapse and reinfection [30]. In contrast, the use of MLMT analysis has proven useful in this respect and allowed to detect a high percentage of relapse cases among the clinical episodes of coinfected patients [4, 5, 25]. In our study, the phylogenetic reconstruction of our data by neighbor-joining (NJ) tree and STRUCTURE analysis was useful to visualize isolate distribution and genetic relationships.

In five patients (P1, P2, P3, P4 and P7), the NJ tree showed clear genetic links between the different isolates; either the genotype was exactly the same or slightly different. The isolates of two patients (P3 and P4) shared the same genotype, showing that these clinical episodes were due to the same *Leishmania* genotype. In these two patients, clinical episodes occurred 6 months after treatment and were not considered as a failure of treatment by clinicians. The close genetic similarity of isolates from the other three patients (P1, P2 and P7) may be due to the evolution of the *Leishmania* population in the patient under pharmacological and/or immunological pressure, and thus these cases could also be interpreted as a relapse. This suggests that in some cases the *Leishmania* population underwent an intrapatient evolution over time. In patients P2 and P7, this hypothesis is supported by the short time between the end of the treatment and the new clinical episode, which was considered by clinicians as a therapeutic failure, unlike the case of patient P1, with 8 and 12 months between clinical episodes.

The isolates of patients P5, P6 and P8, which showed a high dispersion in the NJ tree, suggested another hypothesis. The results obtained with STRUCTURE were globally in agreement with the genetic distance analysis, clearly indicating that isolates from these three patients correspond to different populations and some would be the result of mixing between populations, such as the 6b and 8a samples. These differences cannot be explained only by reinfection events caused by sand fly transmission, since no transmission period occurred between some clinical episodes. A more probable explanation is that of a primo infection with multiple genotypes that were hidden by the culture. Nevertheless, the possible inoculation of different *Leishmania* genotypes by one or several sand flies does not rule out a reinfection by multiple sharing of contaminated syringes between IVDU.

The case of patient P8 is remarkable, with seven isolates, two from clinical episodes and five from non-clinical episodes, represented by 6 different and distant genotypes. Two genetically distant isolates were obtained with only a six-day interval, thus ruling out a reinfection, especially as the patient was hospitalized. Furthermore, this patient developed a second clinical episode, again with a different genotype. This high genetic variability suggests a mixed infection, with a genotype selection during the culture of the samples. Culture bias is a considerable inconvenience for the interpretation of results, as it is impossible to assess if one genotype is more responsible than another for the clinical episode. To avoid culture selection, it would be interesting to work directly with clinical samples, as other authors have done [31].

## Conclusion

The use of multilocus microsatellite markers to analyze isolates taken from *Leishmania*/HIV coinfected patients allowed us to study the evolution of the infections. According to the results and clinical data, we had three different case types in our study: (i) two coinfected patients who relapsed, with the same genotypes in different clinical episodes (25 %); (ii) three coinfected patients who probably relapsed, with slightly different genotypes between clinical episodes due to the evolution of the parasite population within the patient (37.5 %), and (iii) three coinfected patients infected by several distant genotypes in the first clinical episode and a differential selection by the parasite culture performed for the study, or infected over time by non-vectorial transmission associated with IVDU (37.5 %). Due to the chronic nature of leishmaniasis in *Leishmania*/HIV coinfected patients and frequent clinical episode recurrences, close follow-up is required. Our study indicated a great MLMT variability between isolates from patients but, despite this, almost all clinical episodes could be interpreted as relapses.

The results obtained in this research have generated different hypotheses about *Leishmania* parasite behavior in coinfected patients. To explore these hypotheses in more depth, a broader study is required, using a higher number of stocks from coinfected patients monitored over time and from different geographical areas. This study also shows the value of parasite typing to provide new insights into the behavior of *Leishmania* parasites in human.
